# Embedding systematic quality assessments in supportive supervision at primary healthcare level: application of an electronic Tool to Improve Quality of Healthcare in Tanzania

**DOI:** 10.1186/s12913-016-1809-4

**Published:** 2016-10-13

**Authors:** Dominick Mboya, Christopher Mshana, Flora Kessy, Sandra Alba, Christian Lengeler, Sabine Renggli, Bart Vander Plaetse, Mohamed A. Mohamed, Alexander Schulze

**Affiliations:** 1 Ifakara Health Institute, Dar es Salaam/Ifakara, United Republic of Tanzania; 2 KIT Biomedical Research, Royal Tropical Institute, Meibergdreef 39, 1105 AZ Amsterdam, The Netherlands; 3 Swiss Tropical and Public Health Institute, Socinstr. 57, P.O. Box, 4002, Basel, Switzerland; 4 University of Basel, Basel, Switzerland; 5 Novartis Foundation, Novartis Campus, Forum 1-3.92, 4002 Basel, Switzerland; 6 Ministry of Health, Community Development, Gender, Elderly and Children, Samora Avenue, Dar es Salaam, United Republic of Tanzania; 7 Swiss Agency for Development and Cooperation, Freiburgstr. 130, 3003 Berne, Switzerland

**Keywords:** Quality of health services, Quality assessment tool, Supportive supervision, Tanzania, Universal health coverage

## Abstract

**Background:**

Assessing quality of health services, for example through supportive supervision, is essential for strengthening healthcare delivery. Most systematic health facility assessment mechanisms, however, are not suitable for routine supervision. The objective of this study is to describe a quality assessment methodology using an electronic format that can be embedded in supervision activities and conducted by council health staff.

**Methods:**

An electronic Tool to Improve Quality of Healthcare (e-TIQH) was developed to assess the quality of primary healthcare provision. The e-TIQH contains six sub-tools, each covering one quality dimension: infrastructure and equipment of the facility, its management and administration, job expectations, clinical skills of the staff, staff motivation and client satisfaction. As part of supportive supervision, council health staff conduct quality assessments in all primary healthcare facilities in a given council, including observation of clinical consultations and exit interviews with clients. Using a hand-held device, assessors enter data and view results in real time through automated data analysis, permitting immediate feedback to health workers. Based on the results, quality gaps and potential measures to address them are jointly discussed and actions plans developed.

**Results:**

For illustrative purposes, preliminary findings from e-TIQH application are presented from eight councils of Tanzania for the period 2011–2013, with a quality score <75 % classed as ‘unsatisfactory’. Staff motivation (<50 % in all councils) and job expectations (≤50 %) scored lowest of all quality dimensions at baseline. Clinical practice was unsatisfactory in six councils, with more mixed results for availability of infrastructure and equipment, and for administration and management. In contrast, client satisfaction scored surprisingly high. Over time, each council showed a significant overall increase of 3–7 % in mean score, with the most pronounced improvements in staff motivation and job expectations.

**Conclusions:**

Given its comprehensiveness, convenient handling and automated statistical reports, e-TIQH enables council health staff to conduct systematic quality assessments. Therefore e-TIQH may not only contribute to objectively identifying quality gaps, but also to more evidence-based supervision. E-TIQH also provides important information for resource planning. Institutional and financial challenges for implementing e-TIQH on a broader scale need to be addressed.

**Electronic supplementary material:**

The online version of this article (doi:10.1186/s12913-016-1809-4) contains supplementary material, which is available to authorized users.

## Background

Adoption of the Millennium Development Goals (MDGs) as a global framework for action mobilized resources on an unprecedented scale, and resulted in major health gains for many people in low- and middle-income countries. Global improvements in child mortality, and deaths from tuberculosis or malaria, are among the most encouraging results to date [1]. Yet substantial challenges remain, leading to a critical re-appraisal of the MDG framework. One of the most widely-expressed criticisms is that the focus of health-related MDGs on specific diseases and population groups has largely been through vertical strategies, at the expense of more comprehensive measures to strengthen health systems and healthcare delivery [2].

The concept of Universal Health Coverage (UHC) – a prominent sub-target of the health-related Sustainable Development Goal (SDG) – is a broad-based approach. UHC is defined as “*ensuring that all people can use the promotive, preventive, curative, rehabilitative and palliative health services they need, of sufficient quality to be effective, while also ensuring that the use of these services does not expose the user to financial hardship*” [3]. While protection from financial hardship has received most attention, other aspects of UHC such as the quality of health services have been less widely discussed. Consequently, quality of services was described as the missing factor when translating intervention coverage into positive health outcomes. As a result, it was postulated that the third revolution in global health – after those for metrics and accountability – would be a revolution in quality of care [4].

However, there is currently no common understanding of what constitutes ‘quality’ owing to its multi-dimensional and subjective nature. A widely cited definition proposed by a pioneer in work on quality of care is: *“the application of medical science and technology in a manner that maximizes its benefits to health without correspondingly increasing the risk”* [5]. The United States Institute of Medicine defines quality as *“the degree to which health services for individuals and populations increase the likelihood of desired health outcomes and are consistent with current professional knowledge”* [6].

Donabedian’s distinction between structural, procedural and outcome elements is useful when attempting to differentiate between dimensions of quality. Structure refers to physical and staffing characteristics, such as medical staff, supplies, equipment and premises. The procedural element comprises the interactions between users and the healthcare system, i.e. the actual delivery and receipt of care. It involves two types of processes: technical and interpersonal care, defined as *“…technical care refers to the application of clinical medicine to a personal health problem…interpersonal care describes the interaction of healthcare professionals and users or their carers”* [7]. Lastly, outcomes are the consequences of clinical care and the interaction between individual users and the healthcare system. The effectiveness of clinical and interpersonal care determines health status and user satisfaction [7, 8].

Many interventions aimed at improving the quality of care have focused on structural improvements, since these are tangible and relatively easy to achieve. However, evidence indicates that there is only a weak direct link between structural improvements and better health outcomes [9]. According to Campbell et al. [7], this is because structures are only indirect or contingent influencing factors. Structural measures impact on processes, and indirectly on outcomes, since without the necessary skills, supplies and equipment no provider can, for example, carry out an effective examination. However, the limited evidence available suggests that improved quality of care and health outcomes can be achieved more effectively through process changes than through structural measures, even in resource-constrained settings [10]. Hence a number of policy and program interventions focus on process elements [11]. They can be assigned to two categories: measures that *indirectly* influence provider behavior and practice by altering structural conditions (e.g. organization, financing, design of healthcare systems), and interventions that *directly* target the providers. Indirect measures include accreditation programs, targeted retraining, organizational change models, and initiatives to strengthen community participation in health governance and social accountability. Direct measures include peer-review feedback as well as performance-based remuneration and professional recognition [10].

Improving quality requires its accurate measurement. A recent systematic review of health facility assessment mechanisms identified 10 comprehensive tools. Most of the tools focused on health service delivery, especially at primary healthcare level. Healthcare financing and leadership/governance of the health workforce, and some areas of healthcare such as mental health and injury rehabilitation [12], were rarely included. Moreover, it was striking that the majority of these tools were for use in surveys or census-taking and were not routinely applied by regional or council health management teams, for instance in the context of supportive supervision. Yet, systematic identification of quality gaps should be part of supportive supervision as stipulated in the following definition. Accordingly supportive supervision is “…*a process that promotes quality … by … focusing on the identification and resolution of problems, and helping to optimize the allocation of resources … by providing the necessary leadership and support for quality improvement processes and by promoting high standards, teamwork, and better two-way communication*” [13]. If done this way supportive supervision can foster quality improvements [14]. Several approaches and guidelines have been developed to promote supportive supervision, mainly focusing on a specific clinical area, for example malaria case management, reproductive health services or routine immunization services [15]. However, achieving sustained supportive supervision is challenging and must be combined with other measures to effectively improve the quality of service [13, 16–20].

Following development of the first health sector strategic plan in 1999, Tanzania introduced supportive supervision at council level in the early 2000s and has since updated national supportive supervision guidelines on a regular basis [21]. The guidelines state that Council Health Management Teams (CHMTs) are supposed to visit health facilities on a quarterly basis to assess service delivery, share their analysis, then seek solutions with the providers and provide on-site training. However, in the Tanzania Quality Improvement Framework in Health Care 2011–2016, it is stated that such visits are often ineffective in improving quality because supervisors lack time and financial resources, as well as the necessary technical, managerial and supervisory skills, to conduct proper supportive supervision [14, 22]. Further evidence suggests that in general, supervision is often limited to a review of records and medical supplies and negative feedback. It occurs regularly but remains hierarchical, and there is no systematic follow-up in terms of planning and collaborative problem-solving [15, 20, 23]. The goals of the Ministry are therefore to strengthen supportive supervision as well as to ensure more comprehensive monitoring and surveillance.

The “Initiative to Strengthen Affordability and Quality of Healthcare” (ISAQH) program was developed with the aim of informing the expansion of UHC in Tanzania. It includes two key interventions: (1) assessing and improving quality of health services at primary care level as part of supportive supervision and (2) strengthening Community Health Funds (CHFs), i.e. council-based prepayment (insurance) schemes [21, 24, 25]. By early 2015, ISAQH had been rolled out in eight councils. In this article we aim to describe the electronic Tool to Improve Quality of Healthcare (e-TIQH) which is used by CHMTs to assess and foster the quality of health service provision in the context of a broader supportive supervision approach. We focus on the quality assessment methodology and present, for illustrative purposes only, preliminary findings from its application. This paper is the first in a series of forthcoming papers. In these papers we will examine in more depth the trends in quality of health services and the factors driving quality improvements, including the potential effects of e-TIQH on supportive supervision and quality. In this context, we will also compare the e-TIQH-based supervision approach with the conventional routine supervision approach.

## Methods

The quality assessment method described below forms part of a broader supportive supervision approach. Accordingly, data on quality is not collected from research surveys, but is instead gathered by CHMTs in charge of health-related activities. The overall goal of this approach is not only to assess, but also to improve and to maintain quality of primary healthcare provision in resource-constrained settings in a cost effective way, through a three-stage process:

- **Step1:** Assessing the quality of primary healthcare provision in all functioning health facilities with the help of an electronic device, including immediate feedback to healthcare providers and their respective facility governing committee and joint discussions on the causes of quality gaps and possible measures which can be implemented to address those gaps at health facility level.
- **Step 2:** Disseminating the comprehensive assessment findings at council level to health care providers, council authorities and a representative from the regional level as well as developing an action plan to address the identified and jointly discussed quality gaps.
- **Step 3:** Using the assessment findings as additional source for evidence-based planning and budgeting in Council Comprehensive Health Plans (CCHPs), to optimize resource allocation and ultimately quality of health services.

Preliminary results of the use of the e-TIQH were obtained from longitudinal data collected during 2011–2013 from 439 health facilities located in three regions and eight councils of mainland Tanzania: Ulanga, Kilombero, Kilosa/Gairo, Morogoro and Mvomero district councils in Morogoro Region, the Iringa municipal council in Iringa Region as well as Rufiji and Bagamoyo district councils in Coast Region.

### The electronic Tool to Improve Quality of Healthcare (e-TIQH)

Before the introduction of the electronic version of the quality assessment tool in 2011, a paper-based version was used in the two pilot district councils of Ulanga and Kilombero (see Additional file 1). An electronic version was developed, with the same content as the paper version, in order to simplify and make data entry more efficient as well as to automate data analysis. The electronic format also permits immediate and more accurate feedback on results to the health facility staff, so that findings and possible solutions can be discussed at the time of the assessment.

The e-TIQH contains six sub-tools, each covering one essential quality dimension and answering one central question (Table 1).

**Table 1:** The six quality dimensions and respective assessment tools

Sub-tool	Quality dimension	Central question	Assessment tool	Main focus
1	Physical environment and equipment	Do health facilities have sufficient resources and provide a supportive environment to enable providers to fulfill the job expectations that are placed on them?	Checklist	Cleanliness of health facility; availability of equipment and supply; implementation of infection prevention and control (IPC); basic infrastructure of health facility
2	Job expectations	Do providers know what is expected from them in terms of service delivery?	Structured interview and checklist	Knowledge of services provided at the health facility; availability of and knowledge about job descriptions; availability of treatment algorithms and guidelines
3	Professional knowledge and skills	Do health providers have sufficient knowledge and skills to fulfill job expectations?	Direct observation checklist	Adherence to principles of clinical history, physical examination and IPC; management of children under 5 years of age (IMCI), pregnant women, fever patients above 5 years of age and HIV/TB suspects or patients
4	Management and administration of the facility	Do health facilities have a sound management system that provides supportive supervision and feedback to providers and the community?	Checklist	Staffing level; availability of medicines, general patient information, IEC materials and functioning referral system; implementation of record keeping, reporting, mandatory meetings and supervision visits
5	Staff motivation	Are providers motivated to fulfill job expectations?	Structured interview	Participation at trainings and in-house education sessions; implementation of training follow up supervision; timeliness of salary; implementation of promotion scheme; availability of statutory employment benefits
6	Client satisfaction	Are community expectations of health service performance met?	Structured exit-interview	Provision of privacy and courtesy during consultancy, explanations, advice, opportunity to express state of health and ask question

Sub-tools 1, 3 and 4 are checklists, sub-tool 2 is a combination of structured interview and check-list, and sub-tools 5 and 6 are structured interviews for providers and patients. Sub-tool 1 covers items such as the cleanliness and physical infrastructure of the health facility (water and sanitation, waiting and service delivery area, examination room, etc.), implementation of Infection Prevention and Control (IPC) measures and the availability of essential medical equipment and supplies. Sub-tool 2 focuses on the availability of job descriptions and treatment guidelines as well as on providers’ knowledge about their tasks and services to be provided. Sub-tool 3 assesses clinical consultations by means of direct observation, including adherence to the principles of clinical history, physical examination and IPC. It also includes four scenarios for direct observation of consultations with different types of patients: children under 5 years of age (Integrated Management of Childhood Illnesses, IMCI), pregnant women, fever (malaria) patients above 5 years of age, and TB and HIV suspects and patients. Direct observation is considered by the Tanzanian national health authorities an appropriate method for quality control and therefore recommended in the national supportive supervision guidelines [19]. Sub-tool 4 investigates the availability of medicines and supplies, general patient information and Information, Education and Communication (IEC) material. It also captures staffing levels and compliance with record keeping as well as reporting requirements, mandatory meetings and supervision visits at the health facility. Sub-tool 5 examines whether staff have received training, in-house education sessions, training follow up supervision, promotion, regular salary payments and other statutory employment benefits. Based on exit interviews with patients sub-tool 6 captures client satisfaction in terms of patient privacy, staff friendliness, explanations and advice provided by the medical personnel, and opportunity to express state of health and to ask question during consultation.

Each sub-tool contains one or more quality standards accompanied by a set of verification criteria. Standards are qualitative statements defining quality expectations. The criteria are measureable, quantifiable indicators which determine whether the standards have been met. Each criterion is assigned a weight between 1 and 5: 1 indicates a less important criterion, and 5 indicates that the criterion is essential for good quality care.

An example of a quality standard is: “Does the provider adhere to principles of clinical history and physical examination?”, in sub-tool 3. The corresponding verification criteria are “the provider asks open-ended questions”, “the provider systematically performs a physical examination as required on an individual basis”, etc. (Table 2). Each criterion can be answered with either “yes” (value = 1), “no” (value = 0) or “Not Applicable” (NA). A criterion does not apply if the health facility does not have certain tools and infrastructure or delivers specific services.

**Table 2:** Example of the sub-tool structure. Sub-tool 3: Knowledge, skills and ethics of healthcare providers

Indicator	Quality standard to be met	Sub-indicator	Weight	Verification criteria	Score: YES = 1, NO = 0, NA = 99
3.1	Does the provider adhere to principles of clinical history and physical examination?	3.1a	3	The provider greets the client.	
		3.1b	3	The provider sees the client in privacy.	
		3.1c	4	The provider recognizes and addresses non-verbal communication from the client.	
		3.1d	4	The provider asks open ended questions during history taking.	
		3.1e	4	The provider gives the client the opportunity to ask questions, listens and responds.	
		3.1f	4	The provider performs physical examination systematically as per individual case requirement.	
		3.1g	4	The provider requests/performs investigations required and gives clear explanations to the client concerning the purpose of tests and the procedures.	

### Score calculation with the e-TIQH

To determine the quality level for each dimension (sub-tool), a percentage of the maximum possible number of points is calculated. In a first step the average percentage score for each verification criteria is computed, which depends on two factors: the number of responses (healthcare providers or patients interviewed) or direct observations *and* whether the verification criterion was met (i.e. answered with “yes”). In a second step this score is weighted according to the weight which was attributed to the verification criterion in question (1–5). Therefore the total number of points achieved is yielded by dividing the sum of the weights of all average percentage scores per verification criteria by the total number of points achievable. The maximum possible number of points for the respective sub-tools is given in Table 3. In a dispensary one to three providers need to be interviewed with sub-tool 2 and 5, while in hospitals 10 interviews with providers are needed. This approach is flexible and consistent with the purpose of the tool but the amount of data generated necessitates automated data analysis. Finally, the overall quality in a given health facility is calculated as the average percentage score across all six quality dimensions (sub-tools), This score can be used to compare health facilities of a council or even councils and regions, which may be of relevance for resource allocation processes or result-based payments of providers and councils.

**Table 3:** Verification criteria and maximum number of points per quality dimension/sub-tool

Quality dimension/sub-tool	Verification criteria and maximum number of points
1. Infrastructure and equipment of the health facility	41 indicators, 117 points
2. Job expectations	17 indicators, 34 points*
3. Knowledge, skills and ethics	124 indicators, 477 points**
4. Health facility management and administration	33 indicators, 217 points
5. Staff motivation	23 indicators, 66 points*
6. Clients’ satisfaction	6 indicators, 24 points*
	TOTAL: 935 points

*Maximum number of points per provider/patient interviewed

**Maximum number of points if all four clinical scenarios are observed

### Structure and presentation of the e-TIQH

The electronic version was developed by Vodafone Company UK. It comprises a “front end”, i.e. a handheld data collection device (a tablet computer or a smart phone) for the assessors/supervisors, and a “back end”, i.e. a user-friendly dashboard for decision makers with an overview of results, accessible via a laptop, personal computer or a smart phone.

#### Front end

**Fig. 1:**
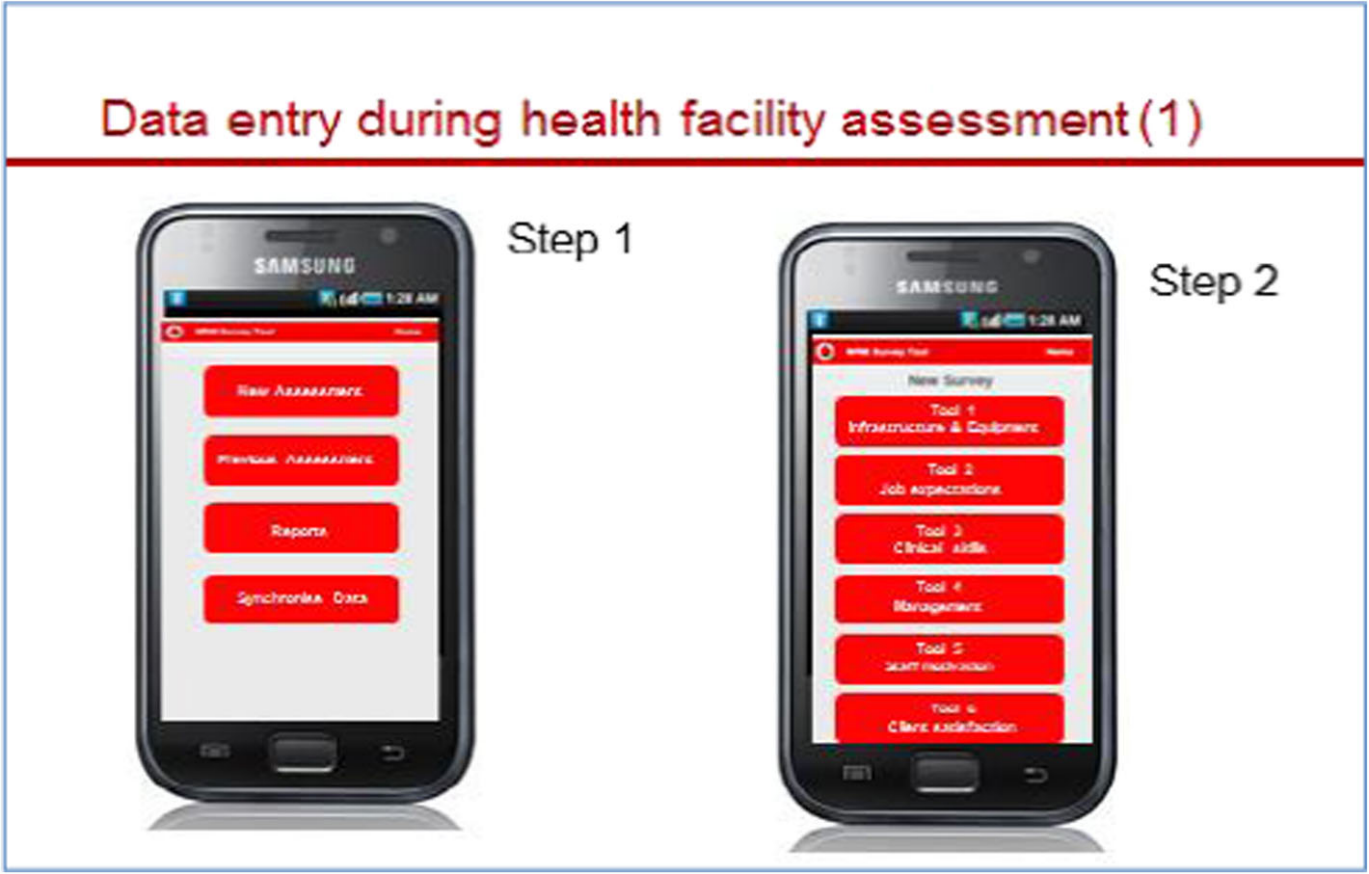
“Front end” of e-TIQH – start pages

**Fig. 2:**
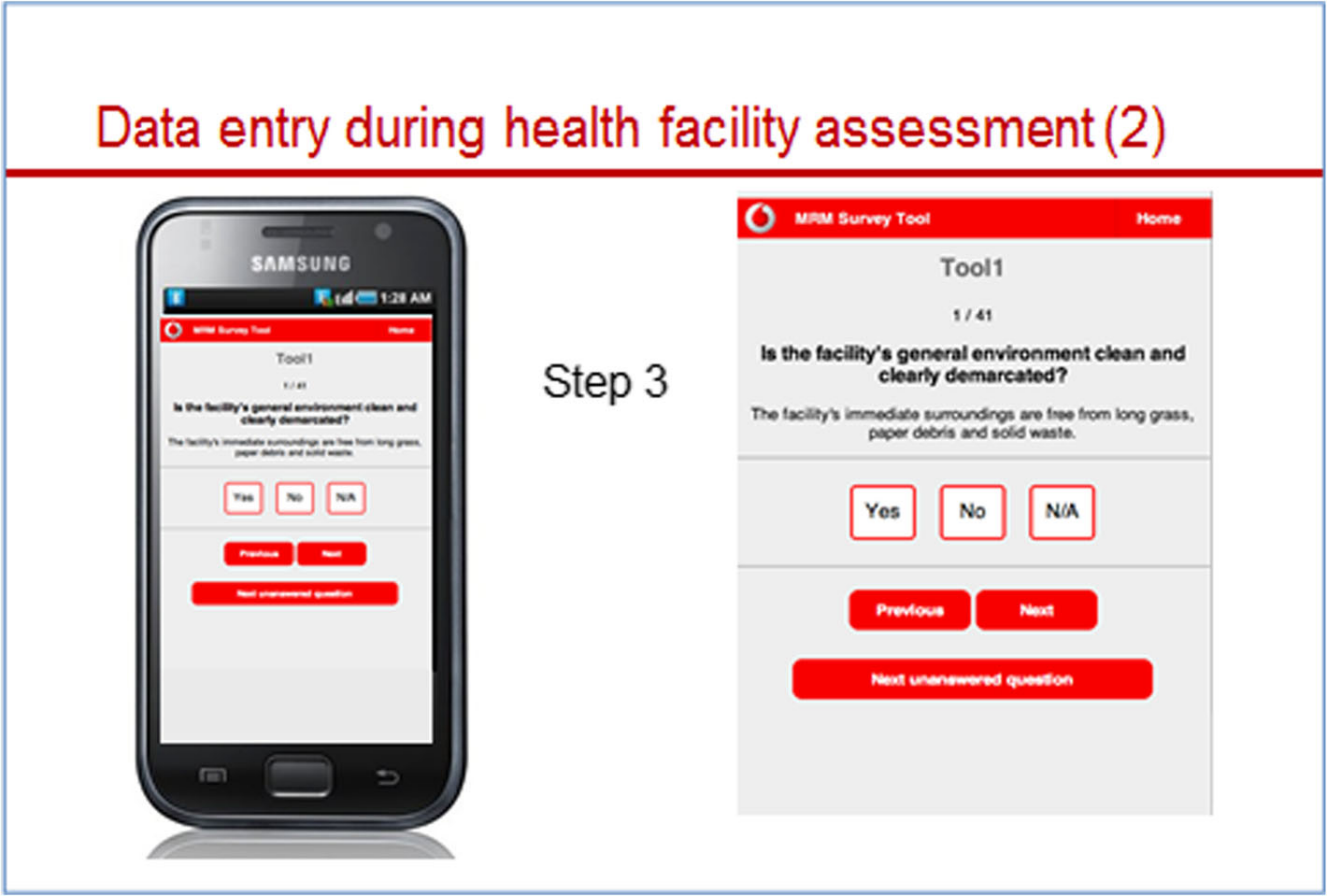
“Front end” of e-TIQH. Only one question displayed on the screen at a time

**Fig. 3:**
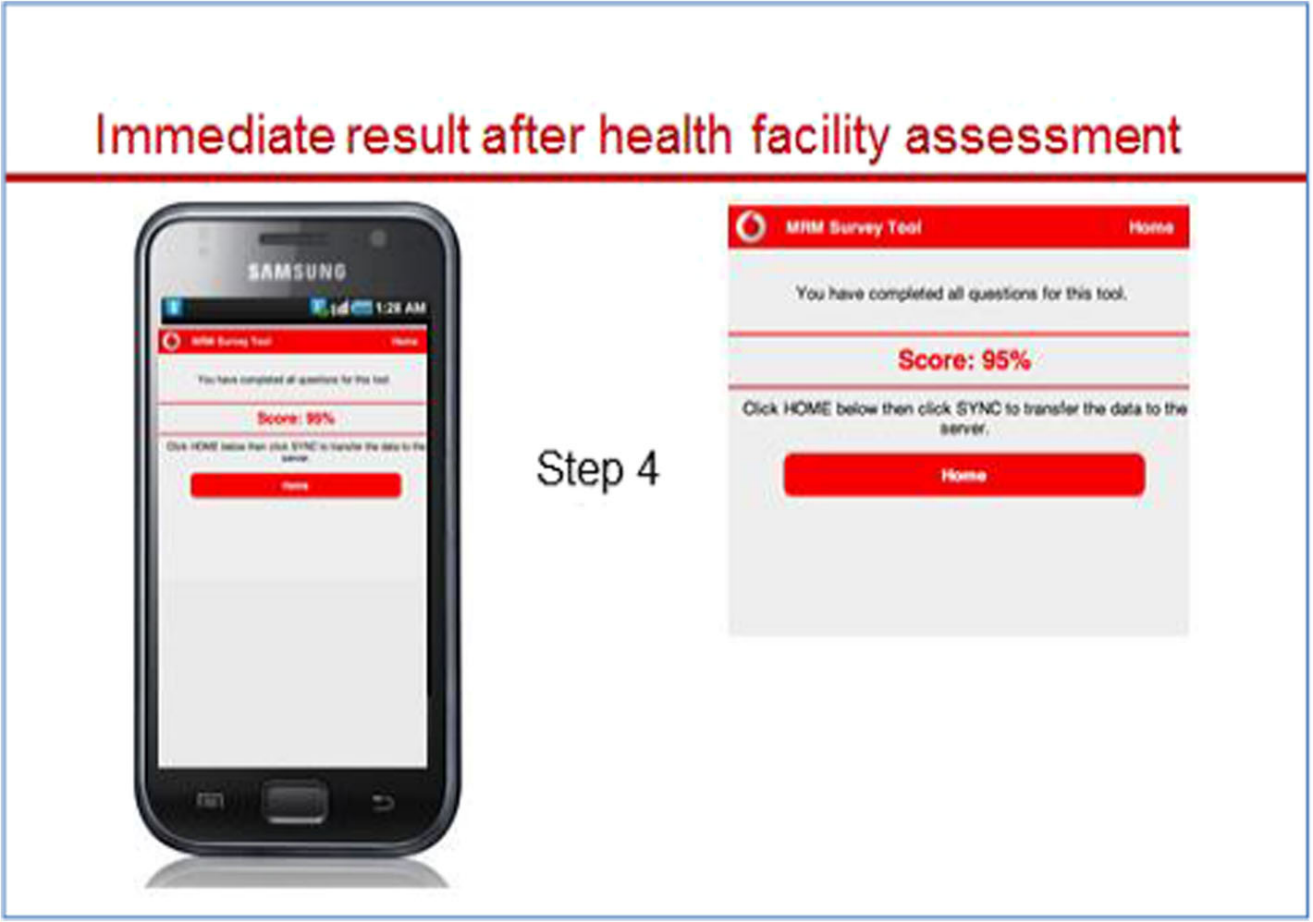
“Front end” of e-TIQH. The score is displayed immediately after assessment

The assessor downloads the assessment tools and stores them on the handheld device. At each assessment, he or she chooses one of the six tools and works systematically through the checklist or questionnaire (Figs. 1 and 2). Once completed, the overall score for the assessment appears (Fig. 3). A list with all verification criteria can also be accessed, whereby criteria marked in green were met and those marked in red were not met. This enables the assessor to give immediate detailed feedback to the provider regarding their own performance or that of the facility, and discuss possible improvement measures.

#### Back end

The system automatically generates statistical reports once the data has been uploaded from the front end device. These can be viewed immediately using a password-protected website by health system managers and decision makers. The following standardized analyses are provided by health facility, council or region:

1. Overall quality across all six dimensions
2. Quality level in each of the six dimensions (Fig. 4) with disaggregated data by verification criterion

**Fig. 4:**
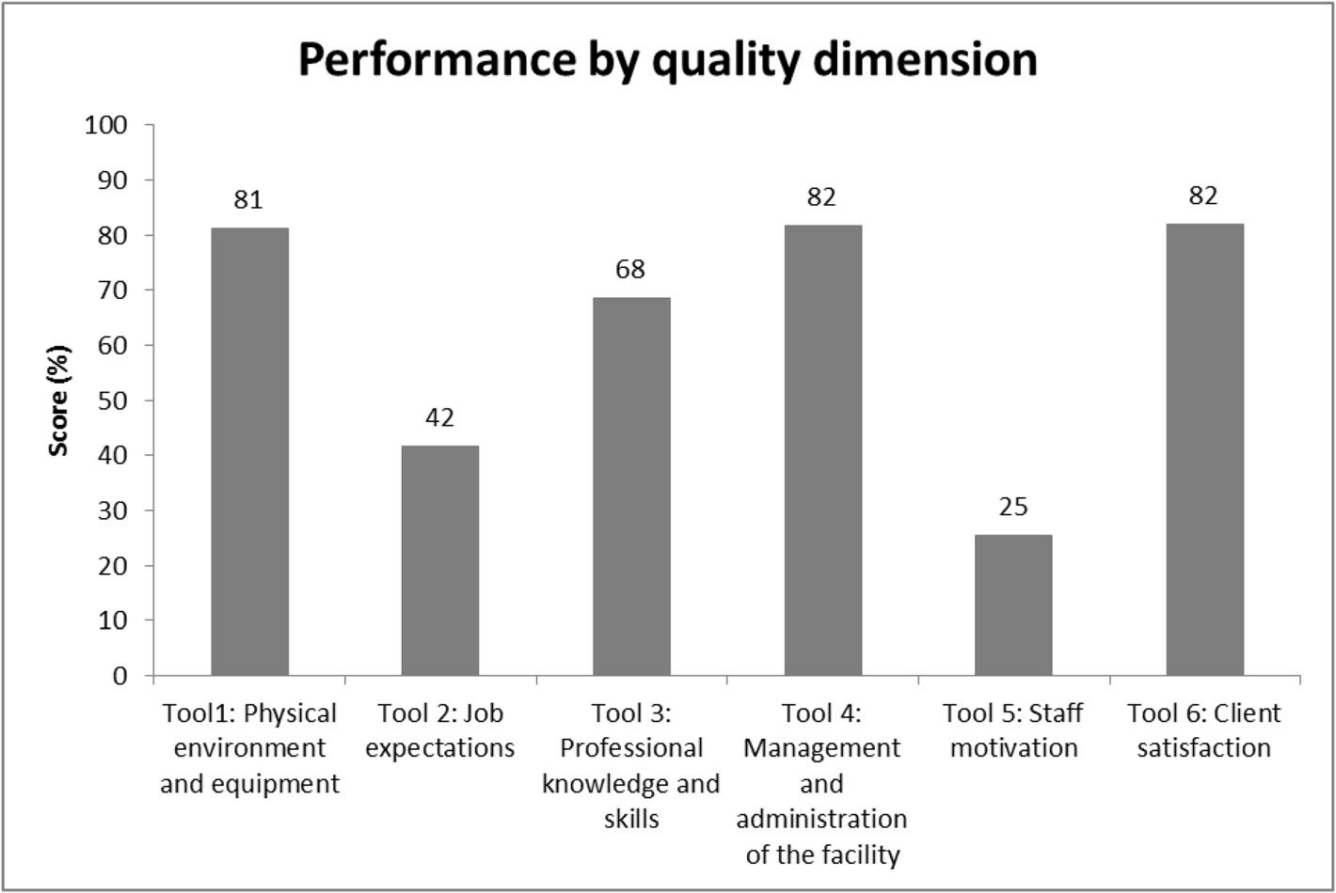
Scores per quality dimension, Iringa council (2012)
3. Quality with regard to disease-specific care (e.g. children under 5 years) (Fig. 5) with disaggregated data by verification criterion

**Fig. 5:**
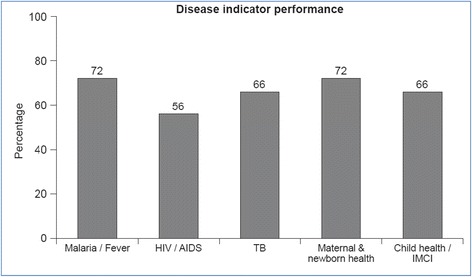
Disease-specific score, Kilosa council (2012)
4. Quality of services by health facility ownership category (faith-based, public, private or institutional)
5. Historical trends for a given health facility, council or region, and for ownership categories or disease-specific care.

### Use of the e-TIQH in the context of supervision activities

The Tanzanian health sector, as other public sectors, is characterized by decentralization by devolution. This principle links decentralization of public service provision e.g. in health and education to devolution of political powers to lower levels as far as possible and feasible. Accordingly, the Tanzanian President’s Office for Regional Administration and Local Government (PO-RALG) oversees and guides the implementation of the policy but local councils have the discretionary power to plan, budget, administer and organize services. At the regional level, Regional Health Management Teams (RHMTs) are strategically positioned to assist the central level in its supervisory and technical support role. RHMTs provide managerial support to Council Health Management Teams (CHMTs) to ensure delivery of quality health services, particularly by conducting routine supportive supervisions to CHMTs. They also have a role in quality improvement of district plans and reports by doing administrative verification after submission of plans and reports by CHMTs and thereafter monitor the progress of the implementation in respective councils within a region. There are various oversight committees at the council level including the Council Social Services Committee (CSSC) which is in charge of education, water and health issues in the council. This committee also oversees the Council Health Services Board (CHSB) which is the governance body overseeing health operations and approving Council Comprehensive Health Plans (CCHPs) and budgets. At operational level, the CHMTs, headed by the District or Municipal Medical Officer (DMO/MMO) are responsible for all health-related activities. The Health Facility Governing Committee (HFGC) oversees the operations at facility level, including the funds generated from cost-sharing arrangements [26].

For the e-TIQH assessment, CHMTs form the core of the assessment teams. However, to maximize objectivity and minimize bias community representatives of the CHSB or the CSSC, providers of private health facilities, as well as selected healthcare professionals from the council, are also assigned to the assessment team. In each council, two teams of six people each conduct annual quality assessments in all primary healthcare facilities (dispensaries, health centers and out-patient departments in council hospitals).

Before the arrival of the assessment team, the chair of the CHMT, who is the District (in the case of a district council) or Municipal (in the case of municipal council) Medical Officer (DMO or MMO) notifies the staff member in charge. Upon arrival, the assessment team leader gives a short overview of the aims and procedures for the visit, either to all staff on duty at a dispensary or health center, or to the medical director, matron(s), hospital administrator(s) and the doctors and nurses in charge of out-patient departments at a hospital. Each of the six members of an assessment team is then assigned to one sub-tool according to his or her expertise and experience. They first observe the physical environment and check the availability of equipment and tools. This is usually recorded by an assessment team health officer in collaboration with the other five team members. They withhold their comments until the health officer has recorded all details. The other five quality areas are then assessed concurrently. Interviews with healthcare providers about job expectations, and direct observations of clinical consultations, require a team member with a medical background. Tool 4 (administration and management of the health facility) and tool 5 (staff motivation) are usually managed by a CHMT member. To complete tool 4, the assessor also receives a list of all essential medicines and supplies which should be available in the health facility. Tool 6 (client satisfaction) is usually managed by the Chairperson of the CHSB or CSSC, most often a Councilor. Depending on the type of health facility, 3–10 medically trained healthcare providers are interviewed with regard to job expectations. The same number of providers (clinicians or nurses) is observed during clinical consultations. Between 5 and 10 trained healthcare providers or patients are interviewed for the assessment of staff motivation and client satisfaction, respectively. Once all the assessors have completed their work, the data are uploaded via a mobile data link to a secure central server. If no internet connection is available, data are stored on the device and uploaded automatically once a connection is established.

### Post-assessment activity

After completion of the assessments, the assessment team assembles in a separate room to compile a summary sheet of the main observations per quality dimension, including strengths and weaknesses. Then, immediate feedback is given to the health providers and the Chairperson of the Health Facility Governing Committee (HFGC). Even without internet access, the assessors can view the score of each checklist or questionnaire and go systematically through it to show the provider which verification criteria were met (marked in green) and which ones were missed (marked in red). The focus of the feedback session is to first identify quality gaps that can be addressed by the providers and their HFGC without support from the council health authorities. Feedback is followed by a discussion of potential solutions to overcome quality gaps, including issues that need to be addressed by the council or higher level. Finally, one copy of the summary sheet is left with the facility manager; the other is kept by the CHMT as a reference for the conventional quarterly supervision visits that do not include quality assessments.

The key results are disseminated to the DMO/MMO and the CHMT. Since the DMO/MMO is registered as a statistics user, he or she can view all results online. Comprehensive findings and possible measures to address the quality gaps are discussed in an annual forum which includes representatives of council authorities (the Council chairperson, selected councilors, the Council Executive Director, the Council Planning Officer, the CHMT and CHSB members), the managers and the HFGC chairpersons of all health facilities in the council, and the owners of private, faith-based and institutional health facilities. A representative from the RHMT and other interested stakeholders such as locally-active NGOs, are also invited. During the forum representatives from the facility level develop an action plan to be implemented at their level, while the council concentrates on measures to be taken at its level. Inputs of all stakeholders are then combined and used for evidence-based planning and budgeting at council level. In councils with a large number of health facilities, the council level forum is followed by zone-based forums to cover all facilities.

### Development and validation of the electronic version of the tool

The tool was developed in two stages. During the first stage (2007–2010), a preliminary paper-based tool introduced by the Ministry of Health, Community Development, Gender, Elderly and Children with support from United States Agency for International Development in the late 1990s and subsequently adapted by the United Nations Population Fund for use elsewhere in Tanzania was field-tested in 2007 in several health facilities of two pilot district councils, Ulanga and Kilombero. After this first test run all quality standards and criteria were reviewed and adapted in consultation with key stakeholders, including clinical experts and representatives from the Ministry as well as regional and council health management teams. This process strictly followed existing national treatment and other guidelines. In the absence of a gold standard against which e-TIQH could be validated, this was considered the best option to ensure validity of the chosen verification criteria. Through unambiguous and clear wording of the verification criteria, additional short explanations for some of the verification criteria and high quality training of the assessors, we strived to allow for reliability of e-TIQH. The tool was then rolled out in all the health facilities in the two pilot district councils, followed by further refinements of the questionnaire and the method of calculating scores per quality dimension. To do this, health facility staff was asked about their experience after each round of interviews, with the aim of identifying missing data. Moreover, the stakeholders agreed on an appropriate weighting system.

During the second stage (2010–2011), the tool was transferred into its electronic format and validated qualitatively and quantitatively using 2010 data, which was available in both the paper-based and electronic formats. For the quantitative part, it was first verified that all quality standards and related criteria were captured in the “front end”. Then, mean scores by council (across all quality dimensions and all health facilities) were compared between the automated analyses of the electronic version and results generated from the paper-based data collection to ensure 100 % consistency.

As part of the qualitative validation of the electronic format, user friendliness of the electronic tool was assessed. After the initial one-day training, assessors navigated without major difficulties through the application, and there were no problems with downloading the application and uploading data. Furthermore, the electronic assessment of a dispensary took on average 1.5 h to complete, compared to 3 h with the paper-based version. Finally, data entry mistakes could be reduced through: 1) a programmed data entry mask (e.g. only one question/criterion visible per electronic page); 2) a “bounce-back” function if a question was not answered; and 3) internal consistency checks.

## Results

Preliminary results are reported here based on data from 2011 to 2013, for illustrative purposes only.

### e-TIQH coverage

The electronic version was first introduced in 2011 in two pilot councils in Tanzania, Ulanga and Kilombero, and was then extended in 2012 and 2013 to a further six councils. In total, these councils include more than 2.5 million people served by 467 health facilities, accounting for approximately 7 % of the country’s health facilities [27]. In 2013, 431 (92 %) of these 467 health facilities were assessed: 14 were hospitals, 43 were health centers and the remainder were dispensaries (Table 4).

**Table 4:** e-TIQH coverage in eight councils of Tanzania, based on the 2013 assessment

	Council		e-TIQH assessment coverage			
Region/Council^a^	Population^b^	No. of health facilities	No. of assessed health facilities^c^	Dispensaries^d^	Health centers	Hospitals
Morogoro Region						
Ulanga DC	265,203	37	37	32	3	2
Kilombero DC	407,880	58	55	49	5	1
Kilosa/Gairo DC	631,186	81	75	64	8	3
Mvomero DC	312,109	63	57	48	6	3
Morogoro DC	286,248	65	55	48	7	0
Iringa Region						
Iringa MC	151,345	28	28	22	4	2
Coast Region						
Bagamoyo DC	311,740	66	64	58	5	1
Rufiji DC	217,274	69	60	53	5	2
Total	2,582,985	467	431	374	43	14

^a^ *DC* District Council, *MC* Municipal Council

^b^United Republic of Tanzania 2012

^c^Out of 467 facilities 36 could not be assessed because the health facility was closed down temporarily by the Ministry of Health and Social Welfare for lack of providers or unsatisfactory infrastructure (14); the health facility was only opened in 2014 (1); facilities were too remote and not reachable by car due to floods or lack of bridges (14) personnel were on leave at the time of assessment (5); access was denied (military base) (2). Not all facilities were assessed in all years

^d^Between the start of the e-TIQH exercise in 2011 and the end of the reporting period in 2013 four dispensaries were upgraded to health centers

### Baseline quality of health service provision

Baseline scores were documented when e-TIQH was introduced (Table 5). A score below 75 % was considered “unsatisfactory”.

**Table 5:** Assessment results by quality dimension (tool) and year, by council (score %)

Council/Year	Tool 1 Infra-structure and equipment	Tool 2 Job expectations	Tool 3 Skills, knowledge and ethics	Tool 4 Administration and management	Tool 5 Staff motivation	Tool 6 Client satisfaction	Mean^a^
Morogoro Region							
Ulanga District Council							
2011 (*n* = 31)	82.1	75.5	87.4	69.9	49.2	93.7	76.4
2012 (*n* = 35)	80.2	76.5	84.8	74.5	65.2	92.6	79.0 ***
2013 (*n* = 37)	78.9	77.7	90.3	75.1	71.7	96.6	81.8 ***
Kilombero District Council							
2011 (*n* = 50)	84.9	61.5	69.9	75.5	45.3	86.9	70.1
2012 (*n* = 51)	84.4	65.3	69.5	84.9	52.9	81.5	73.1 *
2013 (*n* = 55)	80.8	71.2	75.3	82.3	62.9	84.4	76.2 ***
Kilosa/Gairo District Council							
2012 (*n* = 71)	70.2	67.0	74.2	73.3	43.2	77.9	67.7
2013 (*n* = 75)	76.1	70.4	77.8	78.5	49.4	83.9	72.5 ***
Mvomero District Council							
2013 (*n* = 57)	62.8	44.9	65.5	66.3	34.5	77.5	58.5
Morogoro District Council							
2013 (*n* = 55)	58.1	38.0	60.5	59.9	36.0	80.9	55.8
Iringa Region							
Iringa Municipal Council							
2012 (*n* = 25)	81.1	41.5	68.5	81.7	25.4	81.9	63.6
2013 (*n* = 28)	85.1	53.8	80.9	82.4	33.9	85.6	70.3 ***
Coastal Region							
Bagamoyo District Council							
2012 (*n* = 61)	60.7	49.5	77.7	69.3	37.9	81.3	62.7
2013 (*n* = 64)	72.0	55.8	77.0	72.8	41.7	81.1	66.8 ***
Rufiji District Council							
2012 (*n* = 53)	56.5	41.0	61.6	68.8	31.5	68.1	54.6
2013 (*n* = 60)	57.8	49.8	63.9	68.4	34.6	72.1	57.8 **

Note however that the mean difference tested cannot be exactly derived from means reported in the table as it is computed for complete pairs

^a^Asterisks are presented for general orientation purposes and refer to p-values of paired *t*-test comparing mean post-baseline score to mean baseline score: * <0.05, ** <0.01, *** <0.001

Staff motivation and job expectations scored lowest of all quality dimensions. Except for the pilot councils of Ulanga and Kilombero, all other councils scored <45 % for staff motivation. Even in Ulanga and Kilombero, where quality assessments based on an earlier pilot version of the tool had been introduced in 2008, the score at the time of e-TIQH introduction was <50 %. For job expectations, baseline scores did not exceed 50 % other than in the two pilot councils (Kilombero: 61 %; Ulanga: 76 %) and in Kilosa/Gairo (67 %), where quality assessments based on the earlier pilot version began in 2010. Quality with regard to clinical practice (professional skills, knowledge and ethics) of healthcare providers was unsatisfactory in the year of e-TIQH introduction with all councils other than Ulanga and Bagamoyo scoring <75 %. Thus, in these councils at least one in four standard procedures with regard to patient-provider communication, counseling, diagnosis and treatment were not followed by healthcare providers.

Scoring for availability of infrastructure and equipment were relatively low in the rural district councils (56–70 %), but higher in the Iringa municipal council (81 %), an urban council with a smaller number of relatively well-equipped health facilities, and in the two pilot district councils of Ulanga and Kilombero (82–85 %). A similar pattern was seen regarding the administration and management of health facilities: Kilombero (76 %) and Iringa (82 %) scored above the threshold of 75 % while the remainder scored 60–73 %. In contrast, client satisfaction as reported by patients (or their caregivers) during exit interviews was generally high: scores ranged from 68 % in the remote rural district council of Rufiji to 94 % in the pilot district council of Ulanga.

### Trends in quality of health services

Changes over time in the overall quality of health services were assessed in the six councils where at least two consecutive electronic assessments were performed, comparing baseline and post-baseline values by a paired *t*-test. Each council showed a statistically significant increase of 3–7 % in mean score, with the most pronounced improvements in staff motivation and job expectations, the two quality dimensions with the lowest initial score. In Ulanga, Kilombero and Iringa councils, the score for staff motivation increased substantially between 2011 (2012 for Iringa) and 2013 by 23 %, 17 % and nearly 9 %, respectively. However, the absolute score remained at ≤50 % in almost all councils, and no council achieved a satisfactory level (≥75 %). The score for job expectations increased by around 10 % in Kilombero, Iringa and Rufiji but except for Ulanga job expectations remained ‘unsatisfactory’ in all other councils with scores as low as 50 % in Rufiji.

Improvements in clinical practice and facility administration and management were slightly less marked. Iringa municipal council improved its score in clinical practice by more than 10 % within one year (from 68 % in 2012 to 81 % in 2013), while the other councils showed an increase of 2–5 %. Only Bagamoyo remained unchanged at approximately 77 %. Five of the six councils where at least two assessment rounds had been carried out scored above 75 %, with Ulanga reaching 90 %. For health facility administration and management, increases ranged from 4 to 7 %, with the exception of Rufiji and Iringa. Four councils reached a score >75 %, with Iringa and Kilombero achieving the highest scores (82 %).

In terms of the physical environment and availability of functional equipment, increases of around 4–11 % within one year were seen in the Kilosa/Gairo, Iringa and Bagamoyo. Results for this quality dimension were more heterogeneous, however: Rufiji remained at a low score (58 %), and the score for the two pilot district councils of Ulanga and Kilombero declined, though from a high level.

Client satisfaction increased in all councils, except for Kilombero (2011: 87 %; 2013: 84 %), and Bagamoyo, which showed a slight downward trend. Notably, the level of satisfaction in Ulanga was 97 % in 2013.

## Discussion

The e-TIQH assesses a comprehensive range of structural and process aspects of quality in health service provision. The dimensions of infrastructure, equipment, job expectations and facility administration and management mainly contain structural elements, while the areas of professional knowledge, skills and ethics and staff motivation include many procedural aspects. The low score levels in staff motivation observed from preliminary data underline the importance of evaluating the process aspects of quality.

A key element of e-TIQH is that its technology can be applied independently by CHMTs. Experience from the eight participating councils shows that council health staff can handle assessments after proper introductory training and coaching without the help of technical experts. This is mainly because the electronic version includes pre-specified standardized analyses and no data cleaning or analyses have to be performed. E-TIQH reduces the data entry bias and the need for technical and managerial skills which addresses one of the previously stated challenges of routine supportive supervision. Moreover, the technology makes it possible to give real-time feedback which is key to effective mentorship [28].

A second key characteristic of presented methodology which is often lacking in both quality assessment tools and supportive supervision approaches is the evaluation of clinical practice, in the case of e-TIQH through direct observation [12, 14, 29, 30]. Whilst this method has its merits, it also has limitations: the presence of the assessor might lead to changed provider behavior and hence biased data. Standardized patients, often considered as gold standard, and clinical vignettes may measure quality more rigorously and control for case mix, but they do not seem feasible alternatives in the framework of routine supervision. Both methods are relatively expensive and in the case of standardized patients, they are ethically questionable [31]. Another limitation of direct observation especially in low-income settings is that it requires qualified assessors with solid medical expertise in order to do ensure reliability of the method [14]. But even standardized patients method and vignettes require properly trained and instructed observers or interviewers. A validation study comparing direct observation by CHMTs for example with clinical vignettes would generate further evidence on the methodological robustness of e-TIQH.

Moreover, the fact that CHMTs indirectly are assessing their own performance in terms of quality improvements may lead to biased assessments and better results. An accreditation system operating independently from supervision processes, with assessments conducted by national and regional health authorities may help to control potential bias. Another option could be to deploy CHMT members from neighboring councils to assess the health facilities of the council in question. This has been done twice in the two pilot district councils of Ulanga and Kilombero. As a rule, members of CHTMs should not measure quality in those health facilities of which they are otherwise in charge in the frame of supportive supervision.

Finally, as for any supervision or assessment activity, the real challenge for effectively implementing e-TIQH on a broader scale will be institutional and financial sustainability [14]. Supervisors need to be adequately skilled, willing to organize and conduct supervision and facilitate follow-up measures, and have sufficient resources to carry out visits. Council and regional health managers need to consult the evidence which is generated and make use of it in their resource planning. Continuous quality improvement must become part of an organizational culture for both assessors and providers.

The presented results illustrated that the e-TIQH-based analysis provides a fairly comprehensive synopsis of quality gaps. For health facilities mainly located in rural Tanzania, the quality dimensions with the lowest scores were staff motivation and job expectations. This may have contributed to the modest clinical practice observed, and hence presents a threat to quality healthcare provision. Although the standardized statistical e-TIQH reports do not provide evidence on the causes for low staff motivation and job expectations, or determine the drivers for observed improvements, some potentially significant aspects have emerged that merit further research. They can be analyzed in forthcoming papers with the e-TIQH data set. First, anecdotal evidence suggests that quality assessments which are embedded in supervision activities and not solely limited to a review of records and medical supplies may increase staff motivation [14, 32]. An important element for this seems to be the immediate feedback to providers after assessment and subsequent collaborative problem-solving including action plans, especially when it is coupled with consistent follow-up from the CHMTs [33–35]. For instance, council health staff in Kilombero, Ulanga, Iringa and Mvomero have established regular follow-up by phone and physical visits for health facilities with low quality scores to discuss and check progress on agreed improvement measures. Regular follow up supervision, as stipulated in the Tanzanian national supportive supervision guidelines, is essential for the assessments to be of value because health facilities in the eight studied councils have only benefitted from one assessment exercise per year due to resource and time constraints. Addressing the quality gaps identified in the previous assessment round requires some time. However, two assessments per year would be ideal. Another example is a meeting between council health authorities, the project team and healthcare providers from faith-based facilities and church representatives in Ulanga and Kilombero district councils to address low staff motivation in these facilities due to irregular salary payments. In some councils, e-TIQH assessment scores were used by councils on the occasion of World Workers Day to reward providers of the best-performing facilities.

With regard to job expectations, providing missing job descriptions and essential treatment guidelines may have contributed to the increase in scores for this quality dimension. Immediate feedback after direct observation of clinical consultations and targeted on-the-job training (e.g. on infection prevention and control and clinical skills) may have played a role in the positive trend in professional knowledge, skills and ethics observed in health workers in Ulanga, Kilombero, Kilosa/Gairo and Iringa.

Regarding the changing scores for physical environment and equipment, some of the encouraging increases in Kilosa/Gairo, Iringa and Bagamoyo councils could be due to the fact that e-TIQH results informed the council health authorities to budget accordingly in their CCHP. On the other hand, the decreasing scores of the two pilot districts councils Ulanga and Kilombero from a baseline score above 80 % suggest that maintaining infrastructure and equipment over time is a challenge to many health facilities.

A surprising result was the relatively high level of client satisfaction across all councils, contrasting with the low level of staff motivation and the modest score for technical quality of care. This could mean that healthcare providers do not show their frustrations and low motivation when managing patients, or that patients have low expectations with regard to provider behavior. It may also reflect the fact that most patients cannot judge the professional knowledge and skills of healthcare providers, but appreciate the availability of medicines and the friendliness of staff. Methodological reasons may also have contributed: since client satisfaction is assessed through exit interviews conducted near the health facility, clients may not want to disclose their true opinion in case of sanctions from the healthcare provider. Home interviews conducted as part of the community or household survey would be more reliable than exit interviews [36] but are logically unfeasible in the framework of supportive supervision exercises.

The fact that the pilot district councils of Ulanga and Kilombero scored higher in many of the quality dimensions than the rest of the assessed councils may indicate a benefit over time of the e-TIQH-based assessments that are embedded in regular supportive supervision activities. In 2008, these two district councils introduced a paper-based forerunner of e-TIQH and have therefore benefitted from the intervention over a longer period of time.

## Conclusions

The quality of health services must be improved if the goal of UHC in low- and middle-income settings is to be advanced. Extension of service coverage and provision of social health protection for disadvantaged populations will not alone achieve the health-related SDG targets. With the strategic objective of “achieving objectively measurable quality improvement in primary health care services”, the upcoming fourth Tanzanian Health Sector Strategic Plan (2015–2020) embraces this rationale. By linking regular systematic quality assessments to supervision activities, e-TIQH may not only contribute to objectively measuring quality of primary health care, but also to facilitating evidence-based supervision. At the same time, e-TIQH provides important information for resource planning at higher level which is important to address structural quality gaps that cannot be solved at provider level.

The strengths of e-TIQH are its multi-dimensional quality concept and comprehensive data analysis as well as its manageable technology which enables CHMTs to do systematic assessment work and eases its integration in their supportive supervision activities. Immediate structured feedback, discussions on how to address quality gaps and the development of action plans put health workers and HFGCs in an active role to pursue quality improvement.

In terms of planning and budgeting health interventions, e-TIQH can inform the allocation of resources for Council Comprehensive Health Plans, national health sector strategic plans and even national proposals for global financing facilities. If effectively implemented and used, e-TIQH can contribute to more effective decentralization in the health sector by providing an innovative tool to councils for facilitating supportive supervision and improving the quality of healthcare delivery.

## Additional file

Additional file 1: Paper-based version of the e-TIQH assessment tool. (DOC 437 kb)

## Abbreviations

CCHPs: Council Comprehensive Health Plans
CHFs: Community Health Funds
CHMTs: Council Health Management Teams
CHSB: Council Health Services Board
CSSC: Council Social Services Committee
DMO: District Medical Officer
e-TIQH: electronic Tool to Improve Quality of Healthcare
HFGC: Health Facility Governing Committee
IEC: Information, Education and Communication
IMCI: Integrated Management of Childhood Illnesses
IPC: Infection Prevention Control
ISAQH: Initiative to Strengthen Affordability and Quality of Healthcare
MDGs: Millennium Development Goals
MMO: Municipal Medical Officer
NA: Not Applicable
PO-RALG: Tanzanian President’s Office for Regional Administration and Local Government
RHMTs: Regional Health Management Teams
SDG: Sustainable Development Goal
UHC: Universal Health Coverage
