# Supplementary material for: Embedding systematic quality assessments in supportive supervision at primary healthcare level: application of an electronic Tool to Improve Quality of Healthcare in Tanzania
Source: BMC Health Serv Res. 2016 Oct 13;16:578. doi: 10.1186/s12913-016-1809-4 (PMC5064905; doi:10.1186/s12913-016-1809-4)
Supplement: Additional file 1: — Paper-based version of the e-TIQH assessment tool. (DOC 437 kb) [file 12913_2016_1809_MOESM1_ESM.doc]

**TOOL 1: ASSESSMENT OF FACILITY PHYSICAL ENVIRONMENT AND TOOLS/EQUIPMENT**

**Date |__|__|/|__|__|/|__|__|__|__| District Code |__|__|__|**

# DD MM YYYY

**Health Facility Name __________________________________ Health Facility ID |__|__|__|**

**Health Facility Level** |__| **Health Facility Owner** |__|

# 1= Dispensary 1 = Public

# 2= Health Centre 2 = Faith-based

# 3= Hospital 3 = Private for profit

# 4 = Institutional

# Assessor’s name: ___________________________________ Assessor’s Function: _______________________

#

**Directions for use: *Observe all areas of the health facility and enter the score
(either YES = 1, NO = 0 OR NA = Non-applicable = 99) accordingly in the last column.***

| **INDICATOR** | **QUALITY STANDARD TO BE MET** | **SUB-INDICATOR** | **WEIGHT** | | **OPERATIONAL DEFINITION** | | | **SCORE**  **YES=1**  **NO=0**  **NA=99** |
| --- | --- | --- | --- | --- | --- | --- | --- | --- |
| 1.1 | Is the facility’s general environment clean and clearly demarcated? | 1.1a | 1 | | The facility’s immediate surroundings are free from long grass, paper debris and solid waste. | | |  |
|  |  | 1.1b | 1 | | The facility has clear demarcated boundaries. | | |  |
| 1.2 | Does the facility have the required facilities for solid waste management? | 1.2a | 1 | | The facility has a rubbish pit which is properly used and not overflowing. | | |  |
|  |  | 1.2b | 1 | | The facility has solid waste collection equipment, dustbins and trolleys which are properly used and not overflowing. | | |  |
| 1.3 | Are basic facilities available to ensure minimum hygiene at the facility? | **The following basic facilities are available:** | | | | | | |
|  |  | 1.3a | 3 | | The facility has a functional source of clean water supply. | | |  |
|  |  | 1.3b | 3 | | Functional washing points exist in all service delivery points, and soap and water are available. | | |  |
|  |  | 1.3c | 3 | | The facility has a functional waste water drainage system where applicable. | | |  |
|  |  | 1.3d | 3 | | Labelled containers for medical waste disposal are available in all required areas. | | |  |
|  |  | 1.3e | 3 | | The facility has buckets with chlorine solution or other disinfectants to disinfect contaminated instruments in all required areas. | | |  |
|  |  | 1.3f | 3 | | The facility has essential disinfectants and antiseptics. | | |  |
|  |  | 1.3g | 3 | | The facility has a functional incinerator to burn hazardous medical waste which is properly used. | | |  |
|  |  | 1.3h | 3 | | The facility has a placenta pit which is properly used. | | |  |
| 1.4 | Do staff and clients have access to a functioning and clean toilet or latrine? | 1.4a | 3 | | The facility has at least one toilet or latrine for clients and staff. | | |  |
|  |  | 1.4b | 3 | | The toilet or latrine is functional and clean. | | |  |
|  |  | 1.4c | 3 | | Soap and water are available at the washing point near toilet or latrine. | | |  |
| 1.5 | Is the waiting area comfortable for clients? | 1.5a | 1 | | The facility has a comfortable waiting area with enough seats and space for clients. | | |  |
| 1.6 | Is/are the examination room(s) private and comfortable? | 1.6a | 3 | | The examination room(s) ensure(s) privacy. | | |  |
|  |  | 1.6b | 3 | | The examination room(s) has/have a functioning and clean examination couch. | | |  |
| 1.7 | Are facility buildings clean and well lit? | 1.7a | 1 | | All rooms are mopped, free of dust, trash, dirt, spider webs, and the rooms are generally tidy. | | |  |
|  |  | 1.7b | 1 | | All rooms are well ventilated and illuminated. | | |  |
|  |  | 1.7c | 1 | | The facility is free from insects and vermin. | | |  |
|  |  | 1.7d | 1 | | All beds and tables are clean and neatly laid with clean bed sheets and table clothes. | | |  |
| 1.8 | Is an up-to-date inventory list available in each room? | 1.8a | 1 | | Every room has an up-to-date inventory list not older than 6 months. | | |  |
| 1.9 | Does the facility have the following basic/essential medical equipment and supplies*?*  *CHECK EACH ITEM IN THE FACILITY/WARD.*  *INSPECT EACH ITEM TO SEE IF IT IS FUNCTIONING PROPERLY* | **The following essential equipments and supplies are available and functional:** | | | | | | |
|  |  | **Maternal and newborn health:** | | | | | | |
|  |  | 1.9a | 4 | | | Delivery kit where required. |  | |
|  |  | 1.9b | 4 | | | Infant weighing scale where required. |  | |
|  |  | 1.9c | 4 | | | Baby weighing scale where required. |  | |
|  |  | 1.9d | 4 | | | Delivery bed where required. |  | |
|  |  | 1.9e | 4 | | | Partograph where required. |  | |
|  |  | 1.9f | 4 | | | MVA kit where required. |  | |
|  |  | **Other equipments and supplies:** | | | | | | |
|  |  | 1.9g | 4 | | | Stethoscope |  | |
|  |  | 1.9h | 4 | | | Blood pressure cuff/machine |  | |
|  |  | 1.9i | 4 | | | Adult weighing scale |  | |
|  |  | 1.9j | 3 | | | Microscope |  | |
|  |  | 1.9k | 3 | | | Laboratory reagents |  | |
|  |  | 1.9l | 4 | | | RDTm kits where applicable. |  | |
|  |  | 1.9m | 4 | | | HIV test kits |  | |
|  |  | 1.9n | 4 | | | Refrigerator for blood and blood bags where applicable. |  | |
|  |  | 1.9o | 4 | | | Gloves |  | |
|  |  | 1.9p | 4 | | | ENT diagnostic tools (spatula, auriscope, torch) |  | |
|  |  | 1.9q | | 3 | | Thermometer |  | |
|  |  | 1.9r | | 3 | | Wheel chair where applicable. |  | |

***NB: This tool has 9 quality standards and 41 operational definitions. Maximum achievable points are 117.***

**TOOL 2: ASSESSMENT OF JOB EXPECTATIONS**

**Date |__|__|/|__|__|/|__|__|__|__| District Code |__|__|__|**

# DD MM YYYY

**Health Facility Name __________________________________ Health Facility ID |__|__|__|**

**Health Facility Level** |__| **Health Facility Owner** |__|

# 1= Dispensary 1 = Public

# 2= Health Centre 2 = Faith-based

# 3= Hospital 3 = Private for profit

# 4 = Institutional

# Assessor’s name: ___________________________________ Assessor’s Function: _______________________

**Directions for use:**

- ***In dispensaries interview all trained health providers.***
- ***In health centres and hospitals interview 10 health providers.***
- ***Interview providers working in different sections/clinical departments, OPD and IPD.***
- ***Fill out a separate questionnaire for each provider.***
- ***Ask the following questions and enter the score
  (either YES = 1, NO = 0 OR NA = Non-applicable = 99) accordingly in the last column.***

**Interviewee’s job title: |__|__|**

1 = Medical Officer 7 = Enrolled nurse

2 = Assistant Medical Officer 8 = Maternal and Child Health AIDE

3 = Clinical Officer 9 = Medical Attendant

4 = Clinical Assistant 10 = Health Officer

5 = Registered Nurse 11 = Health Assistant

6 = Technician, please specify: ____________

| **INDICATOR** | **QUALITY STANDARD TO BE MET** | **SUB-INDICATOR** | **WEIGHT** | **OPERATIONAL DEFINITION** | **SCORE**  **YES=1**  **NO=0**  **NA=99** |
| --- | --- | --- | --- | --- | --- |
| 2.1 | Can you name at least 5 essential services provided at the facility? | 2.1a | 2 | The provider is able to mention at least 5 essential preventive, promotive and curative services which are provided according to the facility level. |  |
| 2.2 | Do you have a job description and can you mention at least 4 of your key responsibilities? | 2.2a | 2 | The provider has a job description and is able to name 4 key responsibilities stipulated in the job description. |  |
| 2.3 | Do you have the following essential treatment / management guidelines for the different common conditions treated in your facility? | **The provider is able to show the current treatment/management guidelines for**: | | | |
|  |  | 2.3a | 2 | IMCI |  |
|  |  | 2.3b | 2 | Malaria |  |
|  |  | 2.3c | 2 | Opportunistic infections |  |
|  |  | 2.3d | 2 | Sexually Transmitted Infections |  |
|  |  | 2.3e | 2 | Focused Antenatal Care |  |
|  |  | 2.3f | 2 | Post Abortal Care |  |
|  |  | 2.3g | 2 | Emergency Obstetrical Care |  |
|  |  | 2.3h | 2 | Life Saving Skills |  |
|  |  | 2.3i | 2 | TB and Leprosy |  |
|  |  | 2.3j | 2 | Infection Prevention and Control |  |
|  |  | 2.3k | 2 | National Standard Treatment Guideline |  |
| 2.4 | Are the following treatment algorithms for the most common diseases / conditions displayed in the consulting, dispensing and other service delivery areas, in a place where the provider can refer to at a glance? | **The provider is able to show the current treatment algorithms for**: | | | |
|  |  | 2.4a | 2 | IMCI case management algorithm displayed. |  |
|  |  | 2.4b | 2 | ALu treatment algorithm displayed. |  |
|  |  | 2.4c | 2 | IPT algorithm displayed. |  |
|  |  | 2.4d | 2 | Diarrhoea management algorithm displayed. |  |

***NB: This tool has 4 quality standards and 17 operational definitions. Maximum achievable points are 34.***

**TOOL 3: PROFESSIONAL KNOWLEDGE, SKILLS AND ETHICS (PROFESSIONALISM)**

**Date |__|__|/|__|__|/|__|__|__|__| District Code |__|__|__|**

# DD MM YYYY

**Health Facility Name __________________________________ Health Facility ID |__|__|__|**

**Health Facility Level** |__| **Health Facility Owner** |__|

# 1= Dispensary 1 = Public

# 2= Health Centre 2 = Faith-based

# 3= Hospital 3 = Private for profit

# 4 = Institutional

# Assessor’s name: ___________________________________ Assessor’s Function: _______________________

***Directions for use:***

- - - ***Observe once each trained provider in a dispensary and 10 providers in a health centre or hospital.***
    - ***Fill out a separate checklist for each provider.***
    - ***Greet the provider and explain the purpose of the observation.***
    - ***Observe clinical sessions for IMCI, antenatal care, fever case management, or other common conditions treated at the facility.***
    - ***Observe the clinical practice and enter the score (either YES = 1, NO = 0 OR NA = Non-applicable = 99) accordingly in the last column.***

**Interviewee’s job title: |__|__|**

1 = Medical Officer 7 = Enrolled nurse

2 = Assistant Medical Officer 8 = Maternal and Child Health AIDE

3 = Clinical Officer 9 = Medical Attendant

4 = Clinical Assistant 10 = Health Officer

5 = Registered Nurse 11 = Health Assistant

6 = Technician, please specify: ____________

| **INDICATOR** | **QUALITY STANDARD TO BE MET** | **SUB-INDICATOR** | **WEIGHT** | **OPERATIONAL DEFINITION** | **SCORE**  **YES=1**  **NO=0**  **NA=99** |
| --- | --- | --- | --- | --- | --- |
| **3.1** | **Does the provider adhere to principles of clinical history and physical examination?** | 3.1a | 3 | The provider greets the client. |  |
|  |  | 3.1b | 3 | The provider sees the client in privacy. |  |
|  |  | 3.1c | 4 | The provider recognizes and addresses non verbal communication from the client. |  |
|  |  | 3.1d | 4 | The provider asks open ended questions during history taking. |  |
|  |  | 3.1e | 4 | The provider listens and responds to client questions. |  |
|  |  | 3.1f | 4 | The provider performs physical examination systematically as per individual case requirement. |  |
|  |  | 3.1g | 4 | The provider requests / performs investigations required and gives clear explanations to the client concerning the purpose of tests and the procedures. |  |
| **3.2** | **Does the provider apply infection prevention and control measures?** | 3.2a | 4 | The provider washes hands before and after the procedure. |  |
|  |  | 3.2b | 4 | The provider disposes of sharp items in an appropriate way. |  |
|  |  | 3.2c | 4 | The provider puts on gloves where required. |  |
|  |  | 3.2d | 3 | The provider puts on an apron where required. |  |
|  |  | 3.2e | 3 | The provider puts on a mask where required. |  |
|  |  | 3.2f | 3 | The provider puts on boots where required. |  |
|  |  | 3.2g | 3 | The provider puts on goggles where required. |  |

| **INDICATOR** | | **QUALITY STANDARD TO BE MET** | | | **SUB-INDICATOR** | | **WEIGHT** | **OPERATIONAL DEFINITION** | | | **SCORE**  **YES=1**  **NO=0**  **NA=99** |
| --- | --- | --- | --- | --- | --- | --- | --- | --- | --- | --- | --- |
|  | | | | | 3.2h | | 4 | The provider applies proper decontamination procedures by soaking contaminated instruments into a bucket with chlorine or any other disinfectants. | | |  |
| **Scenario A: Observe the IMCI management skills during the assessment of a sick child aged up to 5 years.** | | | | | | | | | | | |
| **3.3** | **Does the provider adhere to IMCI case management process when attending sick children aged up to 5 years?** | | | | **The provider assesses and manages the sick child according to IMCI management processes:** | | | | | | |
|  |  |  |  |  | 3.3a | | 4 | The provider asks if the sick child has had convulsions in the current illness. | | |  |
|  |  |  |  |  | 3.3b | | 4 | The provider asks whether the child vomits everything. | | |  |
|  |  |  |  |  | 3.3c | | 4 | The provider observes whether the child is lethargic or unconscious. | | |  |
|  |  |  |  |  | 3.3d | | 4 | The provider checks if the child is convulsing. | | |  |
|  |  |  |  |  | 3.3e | | 4 | The provider asks about cough or difficult breathing. | | |  |
|  |  |  |  |  | 3.3f | | 4 | The provider asks about diarrhoea. | | |  |
|  |  |  |  |  | 3.3g | | 4 | The provider asks about fever. | | |  |
|  |  |  |  |  | 3.3h | | 4 | The provider asks about ear problems. | | |  |
|  |  |  |  |  | 3.3i | | 4 | The provider assesses the sick child for malnutrition and anemia where appropriate. | | |  |
|  |  |  |  |  | 3.3j | | 4 | The provider assesses the sick child for HIV infection symptoms where indicated. | | |  |
|  |  |  |  |  | 3.3k | | 4 | The provider assesses the sick child for immunization status. | | |  |
|  |  |  |  |  | 3.3l | | 4 | The provider assesses the sick child for other problems. | | |  |
|  |  |  |  |  | 3.3m | | 4 | The provider correctly classifies the sick child. | | |  |
|  |  |  |  |  | 3.3n | | 5 | The provider gives correct treatment for IMCI classification. | | |  |
|  |  |  |  |  | 3.3o | | 5 | The provider demonstrates to mother / caretaker how to administer the medicine to the sick child. | | |  |
|  |  |  |  |  | 3.3p | | 5 | The provider explains to mother / caretaker the danger signs for immediate return. | | |  |
|  |  |  |  |  | 3.3q | | 4 | The provider gives the mother / caretaker a follow up appointment. | | |  |
| **Scenario B: Observe the assessment and management of a pregnant woman.** | | | | | | | | | | | |
| **3.4** | | | **Does the provider adhere to the principles of Focused Antenatal Care during the assessment and management of a pregnant woman?** | **The provider assesses and manages a pregnant woman correctly by enquiring about:** | | | | | | | |
|  |  |  |  | 3.4a | | | 3 | Age | | |  |
|  |  |  |  | 3.4b | | | 4 | Parity | | |  |
|  |  |  |  | 3.4c | | | 3 | Last normal menstrual period | | |  |
|  |  |  |  | 3.4d | | | 3 | History of previous pregnancy | | |  |
|  |  |  |  | 3.4e | | | 3 | Vaginal bleeding /discharge | | |  |
|  |  |  |  | **The provider assesses the following vital signs and other important features:** | | | | | | | |
|  |  |  |  | 3.4f | | 4 | | | Blood pressure | |  |
|  |  |  |  | 3.4g | | 4 | | | Heart rate | |  |
|  |  |  |  | 3.4h | | 3 | | | Respiratory rate | |  |
|  |  |  |  | 3.4i | | 3 | | | Body weight | |  |
|  |  |  |  | 3.4j | | 4 | | | Anaemia | |  |
|  | | |  | 3.4k | | 3 | | | Breasts |  | |
|  |  |  |  | 3.4l | | 3 | | | Oedema |  | |
|  |  |  |  | **The provider examines the pregnant abdomen for:** | | | | | | | |
|  |  |  |  | 3.4m | | 3 | | | Fundal height where required |  | |
|  |  |  |  | 3.4n | | 3 | | | Lie where applicable |  | |
|  |  |  |  | 3.4o | | 3 | | | Presentation where required |  | |
|  |  |  |  | 3.4p | | 3 | | | Fetal heart rate where required |  | |
|  |  |  |  | 3.4q | | 3 | | | Genital |  | |
|  |  |  |  | **The provider requests / performs the following important tests:** | | | | | | | |
|  |  |  |  | 3.4r | | 3 | | | Hemoglobin |  | |
|  |  |  |  | 3.4s | | 3 | | | RPR for syphilis |  | |
|  |  |  |  | 3.4t | | 3 | | | VCT for HIV infection |  | |
|  |  |  |  | **The provider manages and advices a pregnant woman correctly:** | | | | | | | |
|  |  |  |  | 3.4u | | 5 | | | SP, FEFo, Mebendazole and bed net voucher given where required. |  | |
|  |  |  |  | 3.4v | | 5 | | | Danger signs warranting immediate return, i.e. severe headache, fever, vaginal bleeding, blurred vision, difficulties in breathing and abdominal pains, explained to a pregnant woman. |  | |
|  |  |  |  | 3.4w | | 5 | | | Birth preparedness plan, i.e. transport, place of delivery, savings and escort discussed with a client. |  | |
| **Scenario C: Observe the assessment of a fever case in a child above 5 years of age or in an adult:** | | | | | | | | | | | |
| **3.5** | **Does the provider follow the clinical assessment procedures, investigations and treatment guidelines?** | | | | **The provider assesses and performs the following on the patient:** | | | | | | |
|  |  |  |  |  | 3.1a | | 4 | | Detailed history taking including the most frequent possible causes of fever according to age, sex and geographical location of a patient |  | |
|  |  |  |  |  | 3.5b | | 4 | | Body temperature |  | |
|  |  |  |  |  | 3.5c | | 4 | | Anaemia |  | |
|  |  |  |  |  | 3.5d | | 4 | | Jaundice |  | |
|  |  |  |  |  | 3.5e | | 4 | | Enlarged spleen |  | |
|  |  |  |  |  | 3.5f | | 4 | | m-RDT / blood slide where available if malaria is suspected. |  | |
|  |  |  |  |  | 3.5g | | 4 | | Investigations/laboratory tests to determine other causes of fever following clinical judgment. |  | |
|  |  |  |  |  | 3.5h | | 5 | | Clear and correct instructions on how take Alu if malaria is diagnosed. |  | |
|  |  |  |  |  | 3.5i | | 5 | | Instructions on how take medicines prescribed for other diseases / conditions identified. |  | |
|  |  |  |  |  | 3.5j | | 5 | | Advice on prevention of further episodes of the condition / disease treated. |  | |
| **Scenario D: Observe the assessment and management of a (potential) TB / HIV patient where available:** | | | | | | | | | | | |
| **3.6** | **Does the provider assess and manage a (potential) TB / HIV patient correctly?** | | | | **The provider assesses and manages a (potential) TB / HIV patient as per management guidelines.** | | | | | | |
|  |  |  |  |  | **In case of a first contact:** | | | | | | |
|  |  |  |  |  | 3.6a | | 4 | | Main symptoms of TB such as chronic cough, loss of body weight, fever, night sweat, etc. |  | |
|  |  |  |  |  | 3.6b | | 4 | | Chest examination |  | |
|  |  |  |  |  | 3.6c | | 5 | | Sputum examination including clear explanations to the patient on how to collect sputum. |  | |
|  |  |  |  |  | **In case of a positive TB diagnosis:** | | | | | | |
|  |  |  |  |  | 3.6d | | 5 | | Explanations to the patient on DOT regimen and the choice to take the daily treatment either at home or at the health facility, with the help of a treatment supporter or a health worker. |  | |
|  |  |  |  |  | 3.6e | | 5 | | Selection of correct treatment regimen according to body weight. |  | |
|  |  |  |  |  | 3.6f | | 5 | | Clear and correct instructions on daily treatment intake and possible side effects. |  | |
|  |  |  |  |  | **In case of HIV suspect:** | | | | | | |
|  |  |  |  |  | 3.6g | | 5 | | Provider Initiated Counseling and Testing (PICT) session |  | |
|  |  |  |  |  | 3.6h | | 5 | | HIV test based on the patient’s consent. |  | |
|  |  |  |  |  | 3.6i | | 5 | | *If HIV diagnosis is positive:* Clinical staging. |  | |
|  |  |  |  |  | 3.6j | | 5 | | *If indicated:* Antiretroviral treatment. |  | |

|  |  | **In case of home-based TB treatment:** | | | |
| --- | --- | --- | --- | --- | --- |
|  |  | 3.6k | 5 | *If home-based treatment starts:* clear and correct instructions to treatment supporter   - On how to observe daily intake and possible side effects. - On when to collect medicines (once a week during intensive phase, once every two weeks during continuation phase). |  |
|  |  | 3.6l | 5 | *If treatment supporter comes for* *new drugs:*   - Check of drug intake / empty blister packs and side effects. - Provision of correct treatment according to treatment phase. |  |

***NB: This tool has 6 quality standards and 77 operational definitions. Maximum achievable points are 298.***

**TOOL 4: ASSESSMENT OF FACILITY MANAGEMENT AND ADMINISTRATION**

**Date |__|__|/|__|__|/|__|__|__|__| District Code |__|__|__|**

# DD MM YYYY

**Health Facility Name __________________________________ Health Facility ID |__|__|__|**

**Health Facility Level** |__| **Health Facility Owner** |__|

# 1= Dispensary 1 = Public

# 2= Health Centre 2 = Faith-based

# 3= Hospital 3 = Private for profit

# 4 = Institutional

# Assessor’s name: ___________________________________ Assessor’s Function: _______________________

**Directions for use:**

- ***At the facility (hospital, health centre or dispensary) conduct the interview with the health facility in charge, the nurse in charge or the hospital administrator.***
- ***Ask the following questions and enter the score
  (either YES = 1, NO = 0 OR NA = Non-applicable = 99) accordingly in the last column.***

| **INDICATOR** | **QUALITY STANDARD TO BE MET** | **SUB-INDICATOR** | **WEIGHT** | | **OPERATIONAL DEFINITION** | | **SCORE**  **YES=1**  **NO=0**  **NA=99** |
| --- | --- | --- | --- | --- | --- | --- | --- |
| 4.1 | Does the facility have the required staff level and a duty roster? | 4.1a | 5 | | The facility has the required staff level in terms of clinicians and nurses according to its status as dispensary, health centre or hospital. | |  |
|  |  | 4.1b | 3 | | The facility has a duty roster for the staff where applicable. | |  |
| 4.2 | Are the facility opening hours visibly displayed to the public? | 4.2a | 2 | | The opening hours are visibly displayed to the public. | |  |
| 4.3 | Are the currently available services, up-to-date prices and exempted services visibly displayed to the public? | 4.3a | 2 | | Lists of all services and up-to-date prices as well as of exempted services are visibly displayed to the public. | |  |
| 4.4 | Is the clients’ rights chart visibly displayed to the public? | 4.4a | 2 | | The clients rights chart is visibly displayed to the public. | |  |
| 4.5 | Are the following essential IEC materials visibly displayed to clients? | 4.5a | 2 | | IEC materials for malaria visibly displayed to clients. | |  |
|  |  | 4.5b | 2 | | IEC materials for family planning visibly displayed to clients. | |  |
|  |  | 4.5c | 2 | | IEC materials for TB & HIV visibly displayed to clients. | |  |
|  |  | 4.5d | 2 | | IEC materials for STI visibly displayed to clients. | |  |
| 4.6 | Does the facility have mechanisms to get public opinion on the quality of services? | 4.6a | 2 | | The facility has a suggestion box which is used by clients. | |  |
| 4.7 | Product availability | 1. **For how long has SP been available** | | | | | |
|  |  | During the past 90 days or longer | | | | |  |
|  |  | Between 60 and 89 days | | | | |  |
|  |  | Between 30 and 59 days | | | | |  |
|  |  | Less than 30 days | | | | |  |
|  |  | Not applicable | | | | |  |
|  |  | 1. **For how long has Alu Pediatric formulation (dispensable, 5-10 Kgs or 10 to 35Kgs been available?** | | | | | |
|  |  | During the past 90 days or longer | | | | |  |
|  |  | Between 60 and 89 days | | | | |  |
|  |  | Between 30 and 59 days | | | | |  |
|  |  | Less than 30 days | | | | |  |
|  |  | Not applicable | | | | |  |
|  |  | 1. **For how long has Alu Adult formulation (>35Kgs been available:** | | | | | |
|  |  | During the past 90 days or longer | | | | |  |
|  |  | Between 60 and 89 days | | | | |  |
|  |  | Between 30 and 59 days | | | | |  |
|  |  | Less than 30 days | | | | |  |
|  |  | Not applicable | | | | |  |
|  |  | 1. **For how long has Quinine injectables been available?** | | | | | |
|  |  | During the past 90 days or longer | | | | |  |
|  |  | Between 60 and 89 days | | | | |  |
|  |  | Between 30 and 59 days | | | | |  |
|  |  | Less than 30 days | | | | |  |
|  |  | Not applicable | | | | |  |
|  |  | 1. **For how long has Quinine tablets been available?** | | | | | |
|  |  | During the past 90 days or longer | | | | |  |
|  |  | Between 60 and 89 days | | | | |  |
|  |  | Between 30 and 59 days | | | | |  |
|  |  | Less than 30 days | | | | |  |
|  |  | Not applicable | | | | |  |
|  |  | 1. **For how long has Penicillin-G been available?** | | | | | |
|  |  | During the past 90 days or longer | | | | |  |
|  |  | Between 60 and 89 days | | | | |  |
|  |  | Between 30 and 59 days | | | | |  |
|  |  | Less than 30 days | | | | |  |
|  |  | Not applicable | | | | |  |
|  |  | 1. **For how long has Cotrimoxazole been available?** | | | | | |
|  |  | During the past 90 days or longer | | | | |  |
|  |  | Between 60 and 89 days | | | | |  |
|  |  | Between 30 and 59 days | | | | |  |
|  |  | Less than 30 days | | | | |  |
|  |  | Not applicable | | | | |  |
|  |  | 1. **For how long has Cloxacillin been available?** | | | | | |
|  |  | During the past 90 days or longer | | | | |  |
|  |  | Between 60 and 89 days | | | | |  |
|  |  | Between 30 and 59 days | | | | |  |
|  |  | Less than 30 days | | | | |  |
|  |  | Not applicable | | | | |  |
|  |  | 1. **For how long has Amoxycllin been available?** | | | | | |
|  |  | During the past 90 days or longer | | | | |  |
|  |  | Between 60 and 89 days | | | | |  |
|  |  | Between 30 and 59 days | | | | |  |
|  |  | Less than 30 days | | | | |  |
|  |  | Not applicable | | | | |  |
|  |  | 1. **For how long has Metrodinazole been available?** | | | | | |
|  |  | During the past 90 days or longer | | | | |  |
|  |  | Between 60 and 89 days | | | | |  |
|  |  | Between 30 and 59 days | | | | |  |
|  |  | Less than 30 days | | | | |  |
|  |  | Not applicable | | | | |  |
|  |  | 1. **For how long has TB Fixed-dose Combination Therapy for adults in intensive phase FDC been available?** | | | | | |
|  |  | During the past 90 days or longer | | | | |  |
|  |  | Between 60 and 89 days | | | | |  |
|  |  | Between 30 and 59 days | | | | |  |
|  |  | Less than 30 days | | | | |  |
|  |  | Not applicable | | | | |  |
|  |  | 1. **For how long has TB Fixed-dose Combination Therapy for adults in continuation phase FDC been available?** | | | | | |
|  |  | During the past 90 days or longer | | | | |  |
|  |  | Between 60 and 89 days | | | | |  |
|  |  | Between 30 and 59 days | | | | |  |
|  |  | Less than 30 days | | | | |  |
|  |  | Not applicable | | | | |  |
|  |  | 1. **For how long has Zidovudine (AZT) been available?** | | | | | |
|  |  | During the past 90 days or longer | | | | |  |
|  |  | Between 60 and 89 days | | | | |  |
|  |  | Between 30 and 59 days | | | | |  |
|  |  | Less than 30 days | | | | |  |
|  |  | Not applicable | | | | |  |
|  |  | 1. **For how long has Lamivudine (3TC) been available?** | | | | | |
|  |  | During the past 90 days or longer | | | | |  |
|  |  | Between 60 and 89 days | | | | |  |
|  |  | Between 30 and 59 days | | | | |  |
|  |  | Less than 30 days | | | | |  |
|  |  | Not applicable | | | | |  |
|  |  | 1. **For how long has Efaverence (EFV) been available?** | | | | | |
|  |  | During the past 90 days or longer | | | | |  |
|  |  | Between 60 and 89 days | | | | |  |
|  |  | Between 30 and 59 days | | | | |  |
|  |  | Less than 30 days | | | | |  |
|  |  | Not applicable | | | | |  |
|  |  | 1. **For how long has FEFO been available?** | | | | | |
|  |  | During the past 90 days or longer | | | | |  |
|  |  | Between 60 and 89 days | | | | |  |
|  |  | Between 30 and 59 days | | | | |  |
|  |  | Less than 30 days | | | | |  |
|  |  | Not applicable | | | | |  |
|  |  | 1. **For how long has ORS been available?** | | | | | |
|  |  | During the past 90 days or longer | | | | |  |
|  |  | Between 60 and 89 days | | | | |  |
|  |  | Between 30 and 59 days | | | | |  |
|  |  | Less than 30 days | | | | |  |
|  |  | Not applicable | | | | |  |
|  |  | 1. **For how long has Gentamycin been available?** | | | | | |
|  |  | During the past 90 days or longer | | | | |  |
|  |  | Between 60 and 89 days | | | | |  |
|  |  | Between 30 and 59 days | | | | |  |
|  |  | Less than 30 days | | | | |  |
|  |  | Not applicable | | | | |  |
| 4.8 | Does the facility have the essential HIMS books and are they duly filled in? | Less than 30 days | | 2 | | HIMS books are available and duly filled in, and reports are submitted to CHMT. |  |
| 4.9 | Does the Health Facility Governing Committee (HFGC) meet quarterly? | Not applicable | | 3 | | Last quarter HFGC meeting conducted and minutes available. |  |
| 4.10 | Does the facility conduct regular staff meetings to discuss work performance? | 4.10a | | 3 | | Last facility staff meeting conducted and minutes available. |  |
| 4.11 | Does the facility have mechanisms to facilitate referral of emergency patients to the next level? | 4.11a | | 3 | | Provider is able to describe facility plans for referral of patients. |  |
| 4.12 | Has the facility received a supervisory visit in the past 6 months? | 4.12a | | 2 | | The facility has received a supervisory visit in the past 6 months and comments are available. |  |

***NB: This tool has 12 quality standards and 33 operational definitions. Maximum achievable points are 109.***

**TOOL 5: ASSESSMENT OF STAFF MOTIVATION**

**Date |__|__|/|__|__|/|__|__|__|__| District Code |__|__|__|**

# DD MM YYYY

**Health Facility Name _________________________________ Health Facility ID |__|__|__|**

**Health Facility Level** |__| **Health Facility Owner** |__|

# 1= Dispensary 1 = Public

# 2= Health Centre 2 = Faith-based

# 3= Hospital 3 = Private for profit

# 4 = Institutional

# Assessor’s name: ___________________________________ Assessor’s Function: _______________________

**Directions for use:**

- - - ***Interview 5 providers in a dispensary and 10 providers in a health centre or hospital.***
    - ***Fill a separate questionnaire for each provider.***
    - ***Greet the provider and explain the purpose of the interview.***
    - ***Ask the following questions and enter the score
      (either YES = 1, NO = 0 OR NA = Non-applicable = 99) accordingly in the last column.***

**Interviewee’s job title: |__|__|**

1 = Medical Officer 7 = Enrolled nurse

2 = Assistant Medical Officer 8 = Maternal and Child Health AIDE

3 = Clinical Officer 9 = Medical Attendant

4 = Clinical Assistant 10 = Health Officer

5 = Registered Nurse 11 = Health Assistant

6 = Technician, please specify: ____________

| **INDICATOR** | **QUALITY STANDARD TO BE MET** | **SUB-INDICATOR** | **WEIGHT** | **OPERATIONAL DEFINITION** | **SCORE**  **YES=1**  **NO=0**  **NA=99** |
| --- | --- | --- | --- | --- | --- |
| 5.1 | Have you received any in-service training in the previous 3 years? | **The provider has received in-service training in the previous 3 years in the following areas:** | | | |
|  |  | 5.1a | 3 | IMCI |  |
|  |  | 5.1b | 3 | Malaria |  |
|  |  | 5.1c | 3 | VCT |  |
|  |  | 5.1d | 3 | HIV Treatment and Opportunistic Infections |  |
|  |  | 5.1e | 3 | TB and Leprosy |  |
|  |  | 5.1f | 3 | Focused Antenatal Care |  |
|  |  | 5.1g | 3 | Prevention of Mother to Child Transmission |  |
|  |  | 5.1h | 3 | Post Abortal Care |  |
|  |  | 5.1i |  | Family planning |  |
|  |  | 5.1j | 3 | Life saving skills |  |
|  |  | 5.1k | 3 | Immunization |  |
|  |  | 5.1l | 3 | Infection Prevention and Control |  |
| 5.2 | Have you received any supervisory follow up related to the training you attended? | 5.2a | 3 | Supervisory follow up visit received following the training. |  |
| 5.3 | Have you participated in any in-house continuing education sessions in the previous 3 months? | 5.3a | 3 | The provider has participated in in-house continuing education sessions organized by the facility in the past 3 months. |  |
| 5.4 | Have you received your salary on time in the previous 3 months? | 5.4a | 3 | The provider received the salary on time (26^th^-5^th^ of next month). |  |
| 5.5 | Are your promotions effected regularly? | 5.5a | 3 | The provider has been promoted within the previous 3 years. |  |

|  |  | 5.5b | 3 | Salary increment effected following promotion. | |  |
| --- | --- | --- | --- | --- | --- | --- |
| 5.6 | Were you rewarded during the previous 3 years? | **The following types of rewards were given:** | | | | |
|  |  | 5.6a | 3 | Money |  | |
|  |  | 5.6b | 3 | Building materials |  | |
|  |  | 5.6c | 3 | House allocated to entitled staff. |  | |
|  |  | 5.6d | 2 | Appointed as best worker of the year |  | |
|  |  | 5.6e | 2 | Letter of appreciation |  | |
| 5.7 | Are you entitled to a house? | 5.7a | 2 | Other rewards |  | |

***NB: This tool has 7 quality standards and 23 operational definitions. Maximum achievable points are 63.***

**TOOL 6: ASSESSMENT OF CLIENT SATISFACTION**

**Date |__|__|/|__|__|/|__|__|__|__| District Code |__|__|__|**

# DD MM YYYY

**Health Facility Name __________________________________ Health Facility ID |__|__|__|**

**Health Facility Level** |__| **Health Facility Owner** |__|

# 1= Dispensary 1 = Public

# 2= Health Centre 2 = Faith-based

# 3= Hospital 3 = Private for profit

# 4 = Institutional

# Assessor’s name: ___________________________________ Assessor’s Function: _______________________

**Directions for use:**

- - - ***Interview 5 clients in a dispensary and 10 clients in a health centre or hospital.***
    - ***Fill a separate questionnaire for each client.***
    - ***Greet the client and welcome her/him; then explain the purpose of the interview; finally ask for his/her consent and thank her/him.***
    - ***Ask the following questions and enter the score
      (either YES = 1, NO = 0 OR NA = Non-applicable = 99) accordingly in the last column.***

| **INDICATOR** | **QUALITY STANDARD TO BE MET** | **SUB-INDICATOR** | **WEIGHT** | **OPERATIONAL DEFINITION** | **SCORE**  **YES=1**  **NO=0**  **NA=99** |
| --- | --- | --- | --- | --- | --- |
| 6.1 | At the beginning of the consultation, were you given the chance to express your state of health and symptoms? | 6.1a | 4 | The client was given the chance to express her/his state of health. |  |
| 6.2 | During your visit today, did the provider ensure your privacy? | 6.2a | 4 | The client expressed satisfaction with the privacy. |  |
| 6.3 | Did the provider explain the investigations, your health problem and the treatment in clear and simple terms to you? | 6.3a | 4 | The client was given explanations / instructions about the investigations, his/her health problem and treatment. |  |
| 6.4 | During consultation were you given the opportunity to ask questions about the investigations, your health problem and treatment? | 6.4a | 4 | The client was given the opportunity to ask questions about the investigations, his/her health problem and treatment. |  |
| 6.5 | Did the provider listen carefully to your concerns and questions and did he/she give satisfactory answers? | 6.5a | 4 | The provider listened to the concerns and questions of the patient and gave satisfactory answers. |  |
| 6.6 | During consultation, did you get any advice on your health problem? | 6.6a | 4 | Health advice was given during consultation. |  |

***NB: This tool has 6 quality standards and 6 operational definitions. Maximum achievable points are 24.***
